# Alopecia areata universalis nach Sitagliptin-Einnahme: Mögliche immunologische Wirkung von Dipeptidylpeptidase-4-Inhibitoren?

**DOI:** 10.1007/s00105-020-04727-8

**Published:** 2020-11-17

**Authors:** Johannes Kohlmann, Rubén A. Ferrer, Aleksander Markovic, Monica Illes, Manfred Kunz

**Affiliations:** Klinik für Dermatologie, Venerologie und Allergologie, Universität Leipzig, Philipp-Rosenthal-Str. 23, 04103 Leipzig, Deutschland

**Keywords:** Metformin, Dapagliflozin, Diabetes, Haarverlust, Immunsystem, Metformin, Dapagliflozin, Diabetes mellitus, Hair loss, Immune system disorders

## Abstract

Ein 64-jähriger Patient entwickelte 1 Monat nach Therapieeinleitung mit Sitagliptin, einem Dipeptidylpeptidase-4(DPP‑4)-Inhibitor, und Metformin eine Alopecia universalis. Die Therapie des Diabetes wurde auf das Sitagliptin eines anderen Herstellers und Dapagliflozin umgestellt. Auf unser Anraten wurde Sitagliptin abgesetzt und eine Monotherapie mit Dapagliflozin fortgeführt. Nach 6 Wochen war eine erneute Therapie mit Sitagliptin bei unzureichend eingestelltem Diabetes notwendig. Die Alopezie persistierte. Aufgrund des immunologischen Interaktionspotenzials vermuten wir eine Assoziation zwischen DPP-4-Inhibition und der Alopezie. Der kurze therapiefreie Zeitraum scheint zu gering, um ein erneutes Haarwachstum zu beobachten. DPP‑4 kann sowohl eine Inhibition als auch Aktivierung des Immunsystems bewirken.

## Abstract (en)

A 64-year old man developed alopecia universalis after one month of treatment with metformin and sitagliptin, a dipeptidyl peptidase‑4 (DPP-4) inhibitor. Diabetes treatment was changed to another genericum of sitagliptin and dapagliflozin. Following our recommendation, sitagliptin was interrupted and monotherapy with dapagliflozin was continued. After 6 weeks, sitagliptin was reassumed due to unsatisfactory diabetes control. Alopecia did not improve. We suspect a connection between DPP‑4 inhibition and development of alopecia due to its immunological potential. We assume that the treatment interruption might have been too short to induce regrowth of hair. DPP‑4 may result in both inhibition and activation of the immune system.

## Anamnese

Wir berichten über den Fall eines 64-jährigen Patienten mit Erstdiagnose eines Diabetes mellitus Typ 2. Die Initialtherapie erfolgte mit Metformin 1000 mg und dem Dipeptidylpeptidase-4(DPP-4)-Inhibitor Sitagliptin 50 mg täglich. Nach 1 Monat entwickelte der Patient eine Alopecia universalis, die Maximalvariante einer Alopecia areata, mit vollständigem Verlust der gesamten Körper- und Gesichtsbehaarung. Andere Medikamente wurden nach Angaben des Patienten mit Ausnahme von Candesartan im vorangegangenen halben Jahr nicht eingenommen. Vorangegangene Alopezieepisoden wurden verneint, ebenso eine Atopie in der Eigen- oder Familienanamnese. Nach 1 Monat wurde die Diabetestherapie auf 10 mg Dapagliflozin und erneut 50 mg Sitagliptin (anderer Handelsname) jeweils täglich umgestellt. Zwei Monate später stellte sich der Patient in unserer Klinik vor.

## Befund

**Figure 1:**
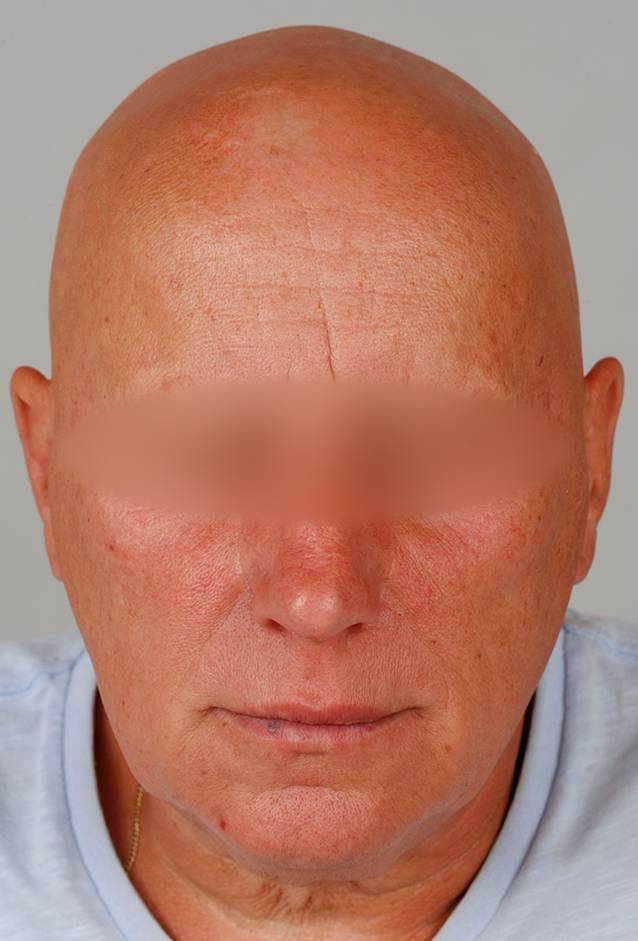

Es zeigte sich ein vollständiger narbenloser Verlust der Körperbehaarung. Exemplarisch ist das Gesicht in Abb. 1 dargestellt. Schilddrüsenparameter, -autoantikörper sowie ANA(antinukleäre Antikörper)-Titer waren normwertig bzw. negativ.

## Diagnose

Alopecia universalis, am ehesten als unerwünschte Arzneimittelwirkung (UAW) auf Sitagliptin.

## Therapie und Verlauf

Aufgrund vorheriger Fallberichte [3, 11] vermuteten wir einen Zusammenhang zwischen der Neueinnahme von Sitagliptin und der Alopezie. Nach unserer Empfehlung erfolgte seitens des Diabetologen eine Umstellung auf eine Monotherapie mit Dapagliflozin. Nach 6 Wochen wurde die Therapie mit Sitagliptin aufgrund eines unzureichend eingestellten Diabetes erneut eingeleitet.

## Beobachtung

Im 6‑wöchigen, therapiefreien Intervall blieb eine Besserung der Alopecia aus. Bis dato ist diese bestehend.

## Diskussion

Die Alopecia universalis wird wegen eines Verlustes des immunologischen Privilegs des Haarfollikels mit konsekutiver autoimmunologischer Inflammation als Autoimmunerkrankung angesehen [10]. Während des Anagens, der Haarwachstumsphase, umzingeln T‑Helfer-Zellen, zytotoxische T‑Zellen, natürliche Killer(NK)- und plasmazytoide dendritische Zellen den unteren Teil des Follikels [1, 13]. In der Vergangenheit wurden genomische Regionen identifiziert, die mit der Alopecia areata assoziiert sind. In diesen Regionen werden unter anderem das zytotoxische T‑Lymphozyten-assoziierte Antigen 4 (CTLA-4), Interleukin(IL)-2, IL-21, IL-2-Rezeptor A und *Eos *(bekannt als *IKZF4*) kodiert [10]. Assoziationen bestehen auch für Gene des Haarfollikels (PRDX5 und STX17) und das ULBP(„cytomegalovirus UL16-binding protein“)-Gen-Cluster. ULBP wiederum aktiviert den NK-Rezeptor-Liganden NKG2D und induziert dadurch möglicherweise eine autoimmunologische Reaktion.

Eine Dysregulation der Immunogenität des Haarfollikels kann grundsätzlich durch bestimmte Zytokine regulatorische Vorgänge des Immunsystems beeinflussen oder einen proinflammatorischen Zustand mit konsekutiver Immunantwort begünstigen.

Sitagliptin, ein DPP-4-Inhibitor, und Metformin, ein Biguanid, sind etablierte Therapieoptionen des Diabetes mellitus Typ 2. Die Naranjo-Wahrscheinlichkeitsskala, ein Punktesystem für die Wahrscheinlichkeit einer UAW, erreicht in unserem Fall für Sitagliptin einen Punktwert von 5/13 und in Bezug auf Metformin 2/13. Es besteht daher eine stärkere Assoziation zwischen der Alopezie und Sitagliptin als kausalem Faktor.

Unsere Literaturrecherche ergab 2 Kasuistiken mit möglicher Assoziation zwischen der Einnahme von Sitagliptin bzw. Metformin und dem Auftreten einer Alopezie. Der erste Fall handelt von einer Patientin, die eine akute Alopezie in Form von Ausdünnen des Haupthaares 3 Monate nach Einnahme von Metformin (Dosierung unbekannt) und einem weiteren Monat mit Dosissteigerung auf 1000 mg 2‑mal täglich entwickelt hatte [3]. Sechs Monate nach Absetzen der Medikation war die Alopezie vollständig regredient. Der zweite Fall handelt von einem Patienten, der initial unter 4‑monatiger Therapie mit Metformin keine UAW zeigte. Vier Monate später, nach Beginn einer kombinierten Therapie mit 50 mg Sitagliptin und 850 mg Metformin, zeigten sich eine Alopezie der Augenbrauen, schnell fortschreitendes Grauwerden des Haupthaars, der Brustbehaarung sowie ein verlangsamtes Bartwachstum [11]. Unter Monotherapie mit Metformin (2850 mg täglich) war die Alopezie 3 Monate später reversibel. Beide Fälle wurden von nichtdermatologischen Fachärzten publiziert und betreut, sodass die klinisch dermatologische Befundbeschreibung nicht eindeutig ist.

Aufgrund der zahlreichen Fixkombinationen aus Gliptinen und Metformin ist eine eindeutige kausale Zuordnung zu einem Auslöser in unserem Fall nicht sicher möglich. Denkbar wäre auch eine Beeinflussung durch Kombination beider Präparate. Aufgrund des autoimmunologischen Interaktionspotenzials vermuten wir jedoch Sitagliptin als mitverantwortlichen Auslöser der Alopezie. Pathophysiologisch kann die Inhibition von DPP‑4 durch Sitagliptin die regulatorische Funktion von DPP‑4 auf T‑Zellen (hier als T‑Zell-Aktivator), stimulierten NK-Zellen, aktivierten B‑Zellen, dendritischen Zellen, Monozyten und Makrophagen stören [5]. DPP‑4 spielt als Korezeptor für CD8^+^-Zellen eine wichtige Rolle bei der erworbenen Immunantwort, der Gedächtnis-T-Zell-Generierung und -Emigration sowie während der Immunseneszenz [5]. Eine Verbindung zu immunologischen Erkrankungen wie Psoriasis, rheumatoide Arthritis, Lupus erythematodes, Sjögren-Syndrom oder dem allergischen Asthma wird beschrieben [5].

Gliptine zeigen teilweise auch ein protektives und immunsuppressives Potenzial. So vermittelt Sitagliptin einen immunsuppressiven Effekt durch Inhibition der T‑Zell-Rezeptor-Signaltransduktion und Proliferation von CD4^+^- und CD8^+^-Zellen [4].

In einem Tiermodell zeigte Linagliptin eine protektive Wirkung bezüglich einer Alopezie [2]. In diesem Modell resultierte die Alopezie aus einem aktivierten Wnt-Signalweg [8], der die Homöostase von Stammzellen negativ beeinflusst. Linagliptin scheint diesen Signalweg zu antagonisieren. Vermutet wird eine Inhibition über die Wnt/β-Catenin-Kaskade, die immunologisch zu einer Aktivierung von dendritischen Zellen mit IL-10-Sekretion und Th1- und Th17-Hemmung führt [12].

Im Gegensatz hierzu werden DPP-4-Inhibitoren ebenfalls mit dem Auftreten eines bullösen Pemphigoids (BP) in Verbindung gebracht [6, 7]. Es wird eine Inhibition von DPP‑8 und DPP‑9 durch Vildagliptin wegen einer geringen Selektivität innerhalb der DPPs vermutet [7]. Dies bewirkt eine konsekutive Aktivierung des Caspase-1-Signalwegs, die zu einem erhöhten Risiko der Entstehung eines BP beisteuert. Andererseits kann DPP‑4 proinflammatorische Zytokine wie TNF (Tumor-Nekrose-Faktor), IL-1β, IL-22, IL-17, IL-23 durch die Peptidaseaktivität deaktivieren [9]. Eine Inhibition kann in einer erhöhten Verfügbarkeit dieser Zytokine resultieren und folglich zu einer Aktivierung einer chronisch inflammatorischen Antwort und/oder Verschlimmerung eines autoimmunologischen Prozesses führen.

Der exakte Einfluss auf Autoimmunologie und immunologische Prozesse ist nicht hinreichend geklärt, da sowohl Aktivierung als auch Inhibition möglich scheinen.

Zusammenfassend vermuten wir in diesem Fall Sitagliptin als auslösenden Faktor für die Alopezie, weil DPP‑4 einen wichtigen Stellenwert hinsichtlich des Immunsystems und der Autoimmunität besitzt. Das therapiefreie Intervall von 6 Wochen vor erneuter Gabe von Sitagliptin erscheint zu kurz, um einen Besserungseffekt zu erzielen. In Fällen, in denen eine immunologische Erkrankung entsteht oder sich verschlimmert, sollte eine Verbindung zu DPP-4-Inhibitoren erwogen werden.

## Fazit für die Praxis

- Neben einer Assoziation zum bullösen Pemphigoid könnten ebenfalls andere autoimmunologische Erkrankungen wie eine Alopezie mit DPP-4(Dipeptidylpeptidase-4)-Inhibitoren in Verbindung stehen.
- Bei Neuauftreten oder Verschlimmerung von vorbestehenden autoimmunologischen Erkrankungen sollte eine Assoziation zu DPP-4-Inhibitoren berücksichtigt werden.
- Der genaue Einfluss (Hemmung oder Aktivierung) von DPP‑4 auf das Immunsystem ist nicht hinreichend geklärt, sodass weitere Untersuchungen sinnvoll erscheinen.
